# Memory Function and Brain Functional Connectivity Adaptations Following Multiple-Modality Exercise and Mind–Motor Training in Older Adults at Risk of Dementia: An Exploratory Sub-Study

**DOI:** 10.3389/fnagi.2020.00022

**Published:** 2020-02-25

**Authors:** Narlon C. Boa Sorte Silva, Lindsay S. Nagamatsu, Dawn P. Gill, Adrian M. Owen, Robert J. Petrella

**Affiliations:** ^1^School of Kinesiology, Faculty of Health Sciences, Western University, London, ON, Canada; ^2^Centre for Studies in Family Medicine, Department of Family Medicine, Schulich School of Medicine and Dentistry, Western University, London, ON, Canada; ^3^Graduate Program in Health and Rehabilitation Sciences, Faculty of Health Sciences, Western University, London, ON, Canada; ^4^School of Health Studies, Faculty of Health Sciences, Western University, London, ON, Canada; ^5^The Brain and Mind Institute, Department of Physiology and Pharmacology, and Psychology, Western University, London, ON, Canada; ^6^Canadian Centre for Activity and Aging, Western University, London, ON, Canada; ^7^Department of Family Practice, Faculty of Medicine, University of British Columbia, Vancouver, BC, Canada

**Keywords:** functional connectivity, functional magnetic resonance imaging, memory, multiple-modality, mind–motor, exercise, cognitive training, older adults

## Abstract

**Background:**

Multiple-modality exercise improves brain function. However, whether task-based brain functional connectivity (FC) following exercise suggests adaptations in preferential brain regions is unclear. The objective of this study was to explore memory function and task-related FC changes following multiple-modality exercise and mind–motor training in older adults with subjective cognitive complaints.

**Methods:**

We performed secondary analysis of memory function data in older adults [*n* = 127, mean age 67.5 (7.3) years, 71% women] randomized to an exercise intervention comprised of 45 min of multiple-modality exercise with additional 15 min of mind-motor training (M4 group, *n* = 63) or an active control group (M2 group, *n* = 64). In total, both groups exercised for 60 min/day, 3 days/week, for 24 weeks. We then conducted exploratory analyses of functional magnetic resonance imaging (fMRI) data collected from a sample of participants from the M4 group [*n* = 9, mean age 67.8 (8.8) years, 8 women] who completed baseline and follow-up task-based fMRI assessment. Four computer-based memory tasks from the Cambridge Brain Sciences cognitive battery (i.e. Monkey Ladder, Spatial Span, Digit Span, Paired Associates) were employed, and participants underwent 5 min of continuous fMRI data collection while completing the tasks. Behavioral data were analyzed using linear mixed models for repeated measures and paired-samples *t*-test. All fMRI data were analyzed using group-level independent component analysis and dual regression procedures, correcting for voxel-wise comparisons.

**Results:**

Our findings indicated that the M4 group showed greater improvements in the Paired Associates tasks compared to the M2 group at 24 weeks [mean difference: 0.47, 95% confidence interval (CI): 0.08 to 0.86, *p* = 0.019]. For our fMRI analysis, dual regression revealed significant decrease in FC co-activation in the right precentral/postcentral gyri after the exercise program during the Spatial Span task (corrected *p* = 0.008), although there was no change in the behavioral task performance. Only trends for changes in FC were found for the other tasks (all corrected *p* < 0.09). In addition, for the Paired Associates task, there was a trend for increased co-activation in the right temporal lobe (Brodmann Area = 38, corrected *p* = 0.07), and left middle frontal temporal gyrus (corrected *p* = 0.06). *Post hoc* analysis exploring voxel FC within each group spatial map confirmed FC activation trends observed from dual regression.

**Conclusion:**

Our findings suggest that multiple modality exercise with mind–motor training resulted in greater improvements in memory compared to an active control group. There were divergent FC adaptations including significant decreased co-activation in the precentral/postcentral gyri during the Spatial Span task. Borderline significant changes during the Paired Associates tasks in FC provided insight into the potential of our intervention to promote improvements in visuospatial memory and impart FC adaptations in brain regions relevant to Alzheimer’s disease risk.

**Clinical Trial Registration:**

The trial was registered in ClinicalTrials.gov in April 2014, Identifier: NCT02136368.

## Introduction

Findings from laboratory work and clinical trials for the treatment of dementias, such as Alzheimer’s disease, have consistently produced disappointing results, with the possibility of a single cure being very unlikely (Mangialasche et al., 2010; Sperling et al., 2011). Efforts have been made to identify and intervene with those who are at greater risk of cognitive decline and dementia before the establishment of clinical impairment (Jessen et al., 2014b). Older adults with subjective cognitive complaints (SCC) (Jessen et al., 2014a; Slot et al., 2018) may represent a portion of the population experiencing early signs of cognitive decline due to underlying pathophysiological changes before clinical impairment is obvious (Chen et al., 2014; Buckley et al., 2015). The focus on preclinical stages of dementia has included the impact of preventive measures such as exercise and cognitive training years prior to disease onset (Livingston et al., 2017). If prevention programs could delay the onset of dementia even in part of the at-risk population, this could decrease the disease prevalence significantly (Brookmeyer et al., 2007; The Alzheimer Society of Canada in collaboration with the Public Health Agency of Canada, 2016). Healthy lifestyle choices, including exercise, may be an important strategy to prevent or slow the progression of dementia in the aging population (Barnes et al., 2003; Alzheimer Society of Canada, 2010; Livingston et al., 2017), even in those with high genetic risk (Lourida et al., 2019).

Exercise has been associated with preserved age-related cognitive functioning in observational studies (Barnes et al., 2003; Abbott et al., 2004; Weuve et al., 2004; Bugg and Head, 2011; Bugg et al., 2012) and improved cognition (Lautenschlager et al., 2008), as well as positive functional (Voss et al., 2010; Chirles et al., 2017) and structural (Erickson et al., 2011) brain changes in longitudinal interventional studies. The positive effects of exercise on behavioral and neuroimaging outcomes in older adults are well documented, but less is known about the effects of exercise in brain functional connectivity (FC). Brain FC can be understood as temporal and functional correlations of spatially distinct cortical and subcortical structures active at rest and/or during task in blood oxygenation level-dependent (BOLD) functional magnetic resonance imaging (fMRI) (Buckner et al., 2009, 2013). Intrinsic FC data consist of anatomically and/or functionally distinct neuronal networks underlying neural function, particularly necessary for higher-order cognitive processes (Buckner et al., 2009, 2013). From a clinical perspective, FC can also aid in the identification of neurodegenerative processes occurring early on in the spectrum of dementia. For instance, Song et al. (2015) reported on resting-state FC disruption in the medial temporal lobe associated with Alzheimer’s disease biomarker deposition in cognitively healthy older adults (Song et al., 2015). Others have postulated that resting-state FC disruption in the default mode network (DMN) is evident in Alzheimer’s disease patients compared to healthy controls (Greicius et al., 2004; Schwindt et al., 2013), which is also pronounced in individuals with mild cognitive impairment (MCI), along with changes in the medial temporal lobe (MTL) network, prior to Alzheimer’s disease diagnosis (Sorg et al., 2007).

Exploring changes in FC in older adults at risk of Alzheimer’s disease and dementia is, therefore, imperative. Of particular interest, previous resting-state fMRI studies have shown that exercise might impart positive effects in enhancing FC in resting-state networks in healthy individuals and in those with MCI (Voss et al., 2010; Chirles et al., 2017). These studies have primarily focused on the effects of aerobic exercise (AE) on FC changes within the DMN and MTL networks in healthy and MCI patients, due to the clinical implications of these networks in the context of Alzheimer’s disease (Greicius et al., 2004; Sorg et al., 2007; Schwindt et al., 2013). Despite promising research with resting-state FC studies, less is known on the effect of multiple-modality exercise on task-related FC in older adults at risk of dementia. Focusing on task-related FC could aid in understanding the influence of exercise in FC underlying neurocognitive processes in those at higher risk of dementia. In addition, as we progress toward more comprehensive interventions that impart improvements to overall health in older adults, it is of interest to investigate whether multiple-modality exercise training (e.g. AE, resistance, or balance training), along with cognitively engaging tasks (i.e. mind–motor training), could have a different impact on FC in these individuals beyond traditional AE alone (Ngandu et al., 2015). Unfortunately, very few studies have explored the effects of combining different exercise modalities (i.e. multiple-modality exercise) and mind–motor training in brain functional and/or structural outcomes (Callisaya et al., 2017; Ji et al., 2017; Rehfeld et al., 2018; Teixeira et al., 2018). Only a short-term (6 weeks), quasi-experimental study included FC as an outcome with results indicating increased FC between the posterior cingulate cortex with cingulate, temporal, parietal, and occipital regions in the multiple-modality exercise group compared to a control group (Ji et al., 2017). Because of limited evidence, further research is warranted.

Square-stepping exercise (SSE) (Shigematsu et al., 2008) is a novel form of mind–motor training, which has been associated with positive effects on global and domain-specific cognitive functioning in older adults (Gill et al., 2016; Boa Sorte Silva et al., 2018b). Although the impact of SSE on cognitive function remains relatively unknown, evidence suggests the potential for SSE to benefit cognition, especially by improving memory (Teixeira et al., 2013; Shigematsu, 2014). Our group has investigated the effects of SSE in cognition, mobility, and oculomotor function in older adults with and without cognitive impairment (Gill et al., 2016; Heath et al., 2017; Boa Sorte Silva et al., 2018a, b). Nevertheless, the effects of SSE on task-related FC remain to be determined.

Therefore, the objective of this exploratory study was to investigate changes in memory function in a group of older adults following multiple-modality exercise with mind–motor training compared to multiple-modality exercise alone. Further, we investigated task-related FC changes in memory in a subsample of older adults with SCC derived from our full randomized controlled trial (RCT) (Boa Sorte Silva et al., 2018b).

## Materials and Methods

### Study Design

Our study design, recruitment, and inclusion criteria have been reported previously (Boa Sorte Silva et al., 2018b). This study is a secondary analysis of memory function outcomes from our full RCT as well as an exploratory study involving a subsample of individuals who underwent fMRI assessment at baseline and 24 weeks. Participants in the experimental group were randomized to a 24-week intervention [*m*ultiple-*m*odality exercise and *m*ind–*m*otor training (M4 group)] targeted at improving cognitive function, mobility, and cardiovascular health (Gregory et al., 2016). Participants in the control group received an active control intervention [multiple-modality exercise plus balance, range of motion, and breathing exercise (M2 group)]. A subsample of participants from the experimental arm (M4 group) underwent fMRI assessment at baseline and 24 weeks later. The study was registered with ClinicalTrials.gov in April 2014 (Identifier: NCT02136368). The Western University Health Sciences Research Ethics Board approved this project, and all participants provided written informed consent prior to taking part in the study.

### Participants

For this secondary analysis of memory function, we examined data from 127 participants, while for the exploratory fMRI study, we examined fMRI data from nine participants who completed both baseline and 24-week assessments. As applied in our full trial (Gregory et al., 2016), the study included community-dwelling individuals aged 55 years or older with self-reported SCC (defined as answering positively to the question “Do you feel like your memory or thinking skills have got worse recently?”) (Barnes et al., 2013), and with preserved instrumental activities of daily living (Gregory et al., 2016). In addition to the full trial inclusion criteria, only right-handed participants were included in this sub-study. Individuals with a diagnosis of dementia and/or scoring <24 on the Mini-Mental State Examination (MMSE) (Gregory et al., 2016), history of stroke or transient ischemic attacks, or presented with MRI contraindications were also excluded.

### Exercise Intervention

The exercise experimental protocol has been published previously (Gregory et al., 2016). Briefly, participants in the M4 group received a 45-min multiple-modality exercise program (i.e. aerobic training and resistance training) with an additional 15 min of mind–motor training (i.e. SSE). Participants in the active control group received 24 weeks of multiple-modality exercise with additional balance, range of motion and breathing exercises. For both arms of the study, sessions were administered in groups of less than 25 participants, 60 min/day, 3 days/week, for 24 weeks.

#### Multiple-Modality Exercise

The multiple-modality exercise intervention incorporated a 5-min warm-up, a 20-min AE, a 5-min cool down, followed by 10 min of resistance training and 5 min of stretching. AE intensity was prescribed via target heart rates (HR) determined at baseline using the STEP^TM^ tool (Stuckey et al., 2012). During the AE component, participants were encouraged to keep their HR at 65–85% of their predicted maximum HR (HRmax) and/or at a rating of 5–8 on the 10-point modified Borg Rating of Perceived Exertion (RPE) scale (Chodzko-Zajko et al., 2009). HR monitoring was conducted part way through and at the end of the AE component during each exercise session. Participants were instructed to record the HR and RPE immediately after each monitoring in a training log provided by the research team. Target HR were recalculated at 12 weeks to adjust for progression in the AE training.

#### Mind–Motor Training

**FIGURE 1:**
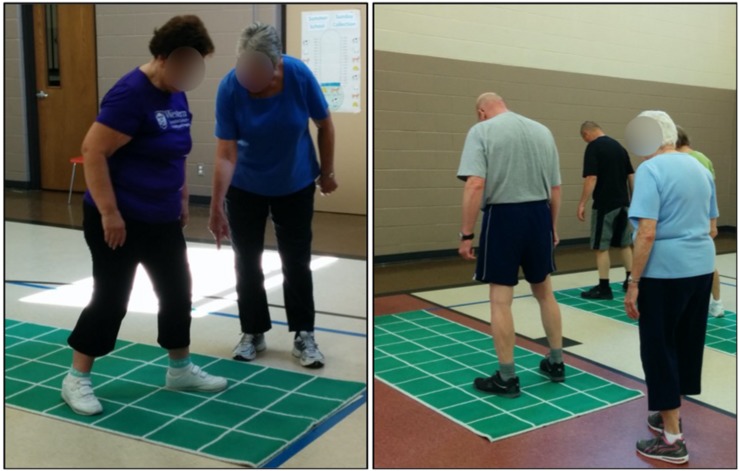
Participants performing stepping patterns during a square-stepping exercise session.

The SSE program is a group-based intervention performed on a gridded floor mat (2.5 × 1 m) containing 10 rows with four equal-sized squares per row. The training protocol entails the reproduction of previously demonstrated complex stepping patterns on the SSE mat (see Figure 1). The stepping patterns are demonstrated by an instructor, and participants are expected to memorize and further attempt to reproduce each stepping pattern by memory. Instructors could not physically intervene, but in instances where participants were having difficulty reproducing the SSE patterns, they were provided oral cues. There are more than 200 stepping patterns created for SSE (Shigematsu et al., 2008), and the complexity of these stepping patterns is given according to the number of steps per pattern, as well as the order and direction of foot placement across the SSE mat. In our study, the SSE sessions were carried out in groups of no more than six participants per mat. To ensure equal group progression throughout the program, the complexity of the stepping patterns within each session was increased only when the majority of participants (i.e. 75%) had successfully performed a given stepping pattern at least four times. The goal was to progress through as many SSE patterns as possible over the 24-week intervention period. Additionally, to create a positive social atmosphere, participants were encouraged to assist each other, as necessary, by providing cues to accurately perform the stepping patterns.

### fMRI Data Collection

Participants were invited to attend a 1-h fMRI session at the Robarts Research Institute at Western University. Image acquisition was performed in a Siemens MAGNETOM Fit whole-body 3 Tesla MRI scanner with in-plane acceleration (GRAPPA = 2). Structural MR images (T1-weighted anatomical images) were acquired for each participant lying passively in the magnet with the following parameters: echo time (TE): 2.98 ms, repetition time (TR): 2,300 ms, time for inversion (TI): 900 ms, and flip angle = 9°, field of view (FOV) = 256 mm, voxel size: 1 × 1 × 1 mm. Whole-brain, task-related functional imaging was performed using a gradient-echo echoplanar imaging (EPI) sequence (36 slices) sensitive to the BOLD contrast with the following parameters: TE: 30 ms, TR: 2,000 ms, flip angle = 70°, FOV = 240 mm, voxel size: 3 × 3 × 3 mm.

The procedure allowed us to acquire 145 functional MR images over 5 min of continuous data collection, while the participants were presented with each cognitive task. Tasks were displayed on a projector screen, visible from the bore of the MRI scanner via a mirror. In each task, participants were required to click on the screen to select their answers using an MRI-compatible tracker ball mouse. The tasks were programmed in the Adobe Flex development environment and were administered as a stand-alone software within the Adobe Integrated Runtime (AIR) environment. The study experiment consisted of a design-free, data-driven approach where a specific design (e.g. block or event-related) was not established (Poldrack et al., 2008; Huettel, 2012). The tasks used have been adapted from tests used in previous neuroimaging and patient studies at our institution (Owen et al., 2010; Hampshire et al., 2012). Tasks were behaviorally piloted by volunteers prior to scanning in order to ensure optimal performance for generating fMRI contrasts of interest (i.e. BOLD). The general approach used for task design was standardized across all four memory tasks described in the subsequent sections.

### Behavioral Tasks

The four cognitive tasks were administered in this study at baseline and 24 weeks and were derived from the Cambridge Brain Sciences (CBS) computerized cognitive battery (Hampshire et al., 2012). Although we collected data from 12 cognitive tasks within the CBS cognitive battery, for this secondary analysis, we decided to focus only on four memory tasks, namely, Monkey Ladder, Spatial Span, Digit Span, and Paired Associates. We had data available from 127 participants at baseline, collected over 2 days using a computer laptop; see our published protocol for more details (Gregory et al., 2016). The rationale to focus on these memory tasks is based on the fact that for our full RCT, the memory composite derived from these four tasks showed trends for greater changes following the 24-week exercise program and showed significant changes 56 weeks after baseline assessments (Boa Sorte Silva et al., 2018b). However, data from each individual task, as well as the fMRI data, have not yet been published. Below is the description of each individual task:

(a) Monkey Ladder is based on a task from the animal literature (non-human primates) and assesses working memory ability (Inoue and Matsuzawa, 2007). In this task, sets of numbered boxes are displayed all at the same time at random locations within a grid. After a variable interval (number of boxes multiplied by 900 ms), the numbers are removed leaving just the blank boxes visible. Participants are requested to respond by clicking on the boxes in ascending numerical sequence. The difficulty of the task is modulated as follows: the number of boxes presented increases by one if the participant answers correctly and decreases by one if the participant makes a mistake. The outcome measure is the length of the longest sequence successfully remembered.
(b) Spatial Span is a task to measure spatial short-term memory capacity in humans (Kessels et al., 2000). In this task, 16 boxes are displayed in a grid. A sequence of randomly selected boxes flashes one at a time at a rate of 900 ms per box. Subsequently, a tone cues the participant to repeat the sequence by clicking on the boxes in the same order in which they flashed. The difficulty of the task is modulated as follows: the number of boxes that flash increases by one if the participant answers correctly and decreases by one if the participant makes a mistake. The outcome measure is the length of the longest sequence successfully remembered.
(c) Digit Span is based on the verbal working memory component of the WAIS-R intelligence test (Wechsler, 1981). In this task, participants view a sequence of digits that appear on the screen one at a time. Subsequently, participants are required to repeat the sequence of numbers using the mouse cursor to click a series of numbered buttons that appear along the bottom of the screen. The difficulty of the task is modulated as follows: the sequence of numbers on the screen increases by one if the participant answers correctly and decreases by one if the participant makes a mistake. The outcome measure is the length of the longest digit sequence successfully remembered.
(d) Paired Associates is a visuospatial paired associate learning task (Gould et al., 2005). In this task, boxes are displayed at random locations on a grid. The boxes open one after another to reveal an enclosed icon, after which they close. Subsequently, the icons are displayed in random order in the center of the grid, and the participant must click on the boxes that contained them. The difficulty of the task is modulated as follows: if the participant remembers all the icon–location pairs correctly, then the next trial will have one more box. If a mistake is made, the next trial has one less box. The outcome measure is the length of the longest sequence successfully remembered.

### Behavioral Data Analysis

All behavioral data collected from our full sample were analyzed using linear mixed models for repeated measurements (Fitzmaurice et al., 2011) to assess differences between groups in mean change from baseline to 24 weeks. In the models, we also examined differences within groups from baseline to 24 weeks. The terms included in the models were group, time, and group × time interaction. Time was modeled categorically using two indicator variables representing each time point (baseline as reference category). Task scores were z transformed. All analyses were performed using the intent-to-treat approach, including all randomized participants, regardless of compliance with the program and follow-up assessments (Fitzmaurice et al., 2011). Behavioral data collected during fMRI image acquisition in our exploratory analysis were analyzed via paired-samples *t*-tests in SPSS®. We also calculated Cohen’s *d* for paired-samples *t*-tests at *post hoc* using the formula $d=t/\sqrt{n}$, where *d* corresponds to Cohen’s *d*, *t* represents *t*-scores, and *n* is the sample size (Lakens, 2013). Analysis of behavioral data was done in order to inform and contextualize the results from fMRI data.

### FMRI Data Analysis

**FIGURE 2:**
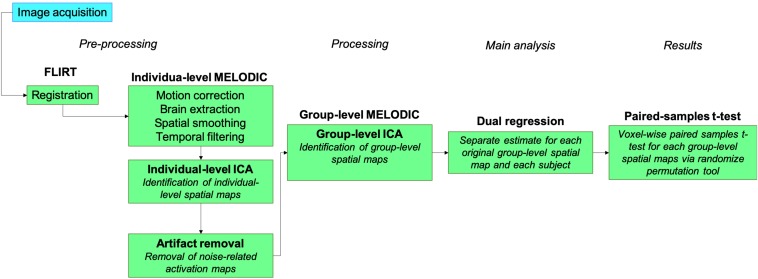
fMRI data analysis pipeline.

All data analysis was performed using FMRIB’s Software Library (FSL) tools.^1^ *Post hoc* analysis was performed in SPSS® for Mac, Version 21 (Armonk, NY, United States). The study pipeline for image acquisition and data analysis is illustrated in Figure 2.

### Preprocessing

Structural images were brain extracted using an in-house script and inspected for optimal extraction. Functional images were registered using FLIRT linear registration to each individual’s structural image and then a 2-mm MNI template registration. We then applied motion correction, brain extraction, spatial smoothing (5-mm FWHM Gaussian kernel) and high-pass temporal filtering (Jenkinson and Smith, 2001; Jenkinson et al., 2002).

### Processing

Functional data analysis was performed using probabilistic independent component analysis (Beckmann and Smith, 2004) as implemented in FSL’s Multivariate Exploratory Linear Decomposition into Independent Components (MELODIC) Version 3.15 (Hyvärinen, 1999; Minka, 2000; Beckmann and Smith, 2004). At the subject level, MELODIC results were decomposed into independent components that represent large-scale patterns of functional network connectivity using independent component analysis (ICA). Individual-level ICA maps were inspected to identify components that were considered noise using a visually inspected structured artifact removal approach (i.e. hand removal) (Griffanti et al., 2017), as previously applied in a similar exercise study (Rajab et al., 2014). All independent components that were identified as noise were removed from individual-level data via spatial regression using FSL’s *fsl_regfilt* tool. These components were composed of noise due to several sources such as head motion, cerebral spinal fluid signal, respiratory and cardiac rhythms, scan parameters, and others.

### Main Analysis

Following individual-level MELODIC, we then performed group-level ICA to identify independent components that represent large-scale patterns of FC within the group-level spatial maps, and the independent components were set at 40 per task, based on inspection of individual-level ICA results to inform optimal fitting of the data. Results from group-level MELODIC were further analyzed using FSL’s dual regression tool. In this approach, the set of spatial maps from the group-average analysis was used to generate subject-specific versions of the spatial maps, and associated time series (Nickerson et al., 2017). Primarily, for each individual in the study, the group-average set of spatial maps is regressed (as spatial regressors in a multiple regression) into the subject’s 4D space–time dataset; this results in a set of subject-specific time series, one per group-level spatial map. Later, those time series are regressed (as temporal regressors in a multiple regression) into the same 4D dataset, resulting in a set of subject-specific spatial maps, one per group-level spatial map. This procedure ultimately unfolds in a separate estimate for each original group-ICA map and each subject. In the final step of our analysis, we then performed paired-samples *t*-tests in a voxel-wise analysis for each of the group-level spatial maps using FSL’s randomize permutation-testing tool (5,000 permutations, threshold-free cluster enhancement) corrected for voxel-wise multiple comparisons. Our goal was to identify any significant changes in the group-level spatial maps from baseline to 24 weeks. If any changes were identified, our results could indicate that the exercise program might have imparted adaptations in FC.

### *Post hoc* Analysis

We further performed *post hoc* analysis using subject-specific spatial maps (stage 2 outputs from dual regression) to quantify changes in the strength of connectivity within a group-level spatial map from baseline to 24 weeks, following previous methodology (Glahn et al., 2010). To accomplish this, we used the group-level spatial map as binary network masks and calculated an index that would indicate, on average, how strongly the voxels within a group-level spatial map are related to each other for each individual (via FSL’s *fslmeants*). We were interested in knowing whether this FC index would have changed following the exercise program (Glahn et al., 2010; Rajab et al., 2014). We also performed a similar procedure to quantify the changes in specific regions that showed significant changes from baseline to 24 weeks in the main analysis. Instead of using a binary mask, this was accomplished by extracting a voxel connectivity index from the exact location where changes from baseline to 24 weeks occurred (i.e. using MNI152 coordinates in *fslmeants*); the coordinates were defined based on significant or borderline significant results of dual regression. The indices calculated as a result of these procedures were then analyzed in a paired-samples *t*-test in SPSS®.

## Results

Details regarding study enrolment, randomization, and adherence have been reported elsewhere (Boa Sorte Silva et al., 2018a, b). Briefly, 169 individuals were assessed for eligibility, 11 did not meet the inclusion criteria, and 31 declined to participate. Thus, 127 participants were included and randomized to either the M2 (*n* = 64) or M4 (*n* = 63) groups; 109 participants attended assessments at 24 weeks. Demographic characteristics for our full sample are shown in Table 1. For our fMRI exploratory study, the sample was composed of mostly females who were approximately 70 years of age and with a Montreal Cognitive Assessment (MoCA) score of approximately 25, suggesting the presence of objective cognitive impairment in addition to the self-reported SCC but with no indication of dementia (mean MMSE score of 29) (Nasreddine et al., 2005). Participant demographic and clinical characteristics for this subsample are presented in Supplementary Table 1.

**TABLE 1:** Baseline characteristics of study participants by randomization group.

**Variables^†^**	**M4 (*n* = 63)**	**M2 (*n* = 64)**
**Demographics**		
Age, year	67.6 (7.5)	67.4 (7.2)
Women	44 (69.8%)	46 (71.9%)
Caucasian	61 (96.8%)	62 (98.4%)
Education, year	13.3 (2.7)	13.8 (3)
MoCA, score	25.3 (2.7)	25.6 (2.4)
MMSE, score	29 (1.2)	29.2 (1)
Weight, kg	80 (13.8)	80.8 (17.7)
Height, m	1.65 (0.1)	1.65 (0.1)
BMI, kg/m^2^	29 (4.1)	29.7 (6.2)
**Medical history, *n* (%)**		
Hypertension	36 (57.1%)	32 (50%)
Hypercholesterolemia	28 (44.4%)	23 (35.9%)
Type 2 diabetes	7 (11.1%)	5 (7.8%)
Myocardial infarction	5 (7.9%)	4 (6.3%)
Atrial fibrillation	3 (4.8%)	–
Angina/coronary artery disease	2 (3.2%)	1 (1.6%)
Aneurysm	2 (3.2%)	1 (1.6%)
Former smoker	29 (46%)	28 (44.4%)
Current smoker	1 (1.6%)	1 (1.6%)
**Memory Tasks, *z* scores**		
Monkey Ladder	0.05 (1.03)	−0.05 (0.97)
Spatial Span	−0.04 (1.05)	0.04 (0.95)
Digit Span	−0.1 (1.03)	0.28 (1.75)
Paired Associates	−0.09 (0.95)	0.09 (1.05)

*^†^Data presented either as mean (standard deviation) or no. (%) where applicable. M2, multiple-modality group; M4, multiple-modality, mind–motor group; MMSE, Mini-Mental State Examination; MoCA, Montreal Cognitive Assessment; BMI, body mass index.*

### Behavioral Results

For our full sample (*n* = 127), the M4 group showed greater improvements in the Paired Associates tasks compared to the M2 group at 24 weeks [mean difference: 0.47, 95% confidence interval (CI): 0.08 to 0.86, *p* = 0.019] (see Table 2), which resulted from an improvement in the M4 group from baseline to 24 weeks (*p* = 0.001), while changes in the M2 group were not observed (*p* = 0.93). Participants in both groups showed improvements in the Monkey Ladder task (*p* ≤ 0.01); however, there were no differences between groups at follow-up. No within- or between-group changes were observed for the Spatial Span and Digit Span tasks; however, the M4 group showed trends for improvements in the Digit Span task (*p* = 0.06).

**TABLE 2:** Within- and between-group differences from baseline to 24 weeks by randomization group.^†^

	**Within-group differences (95% CI)**				**Between-group differences (95% CI)**	
**Outcomes**	**M4 (*n* = 63)**	***p***-**value**	**M2 (*n* = 64)**	***p*-value**	**24 weeks (*n* = 127)**	***p*-value**
Monkey Ladder	**0.23 (0.05 to 0.41)**	**0.01**	**0.29 (0.12 to 0.47)**	**0.001**	−0.07 (−0.32 to 0.19)	0.6
Spatial Span	−0.07 (−0.25 to 0.12)	0.47	0.04 (−0.14 to 0.22)	0.67	−0.11 (−0.36 to 0.15)	0.42
Digit Span	0.33 (−0.02 to 0.69)	0.06	−0.06 (−0.4 to 0.29)	0.75	0.39 (−0.1 to 0.88)	0.12
Paired Associates	**0.48 (0.2 to 0.76)**	**0.001**	0.01 (−0.26 to 0.28)	0.93	**0.47 (0.08 to 0.86)**	**0.019**

*^†^Calculated from linear mixed effects regression models that included group (M4 or M4), time (baseline and 24 weeks), and group × time interaction terms. A total of four models were conducted—corresponding to each memory task listed in the first column. Results are represented as intent-to-treat approach. Bold numbers indicate significant differences within- or between-groups where applicable. M2, multiple-modality group; M4, multiple-modality, mind–motor group.*

For our subsample of participants in the fMRI exploratory study (*n* = 9), the results indicated no significant differences from baseline to 24 weeks in all of the tasks studied. For the Paired Associates task, however, we observed a trend for significant differences compared to baseline for the task max score [mean difference: 0.75, 95% CI: −0.1 to 1.6, *t*(7) = 2.05, *p* = 0.08, Cohen’s *d* = 0.72] and task mean score [mean difference: 0.4, 95% CI: −0.1 to 0.8, *t*(7) = 0.08, Cohen’s *d* = 0.74], corroborating the results from our full sample. The results are presented in Supplementary Table 2.

### fMRI Results

Group-level ICA via MELODIC identified several independent components across all four tasks; one component included previously studied networks such as the DMN (Supplementary Figure 1). Considering the exploratory nature of the study, we investigated significant and borderline significant changes across all independent components identified across all four tasks. Dual regression results indicated significant change in FC after the 24-week program within only one of the group-level spatial maps in the Spatial Span task and overall borderline significant changes in eight other regions in the brain, of which seven were further explored and one was excluded as it was considered not relevant for the purposes of this study. The results for each task are reported in further detail below, except for the Monkey Ladder task as no differences were observed at post-test.

**FIGURE 3:**
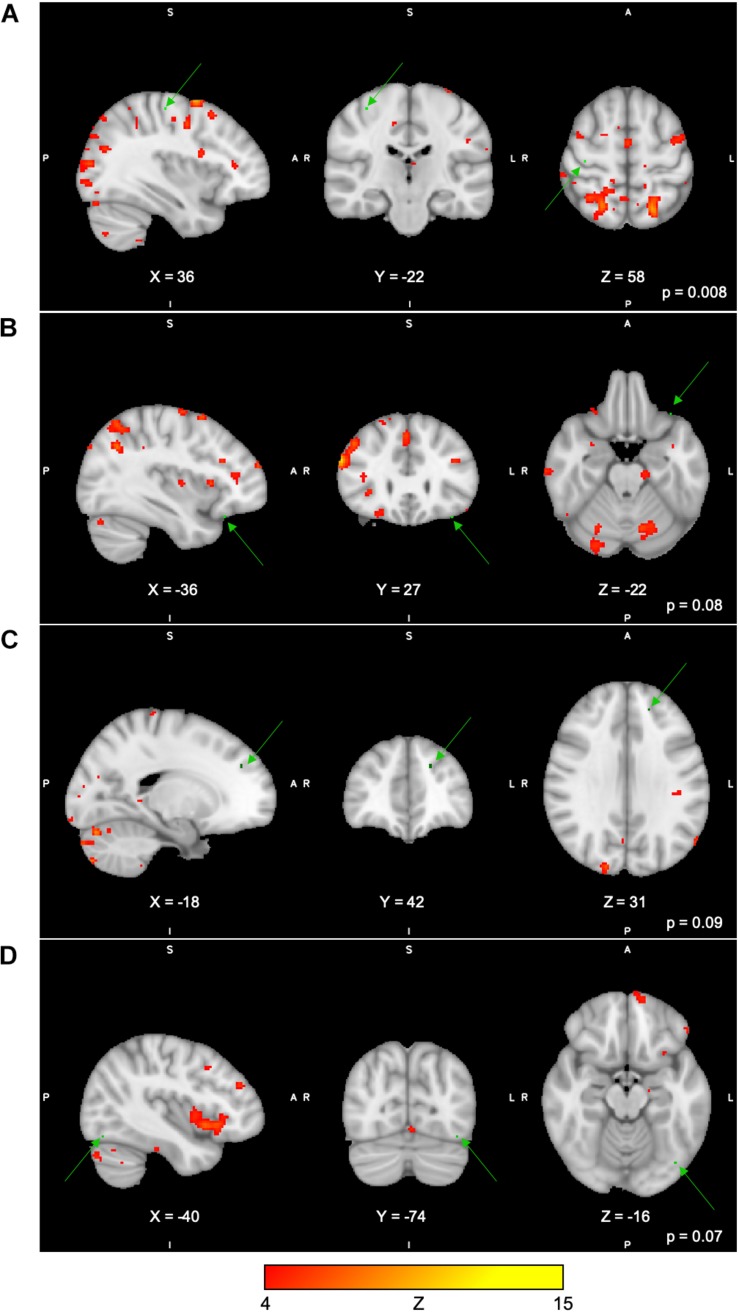
**(A–D)** Changes within group during Spatial Span task. In red–yellow contrast are Spatial Span-independent components 16 **(A)**, 06 **(B)**, 23 **(C)**, and 30 **(D)**. In dark–light green are regions with changes in group-level spatial map co-activation after the exercise program (green arrows).

For the Spatial Span task across all 40 group-level spatial maps [i.e. Spatial Span-independent components (SS)], dual regression revealed significantly decreased co-activation in the right precentral/postcentral gyri (MNI: 36, −22, 58) after the exercise program within SS16 (corrected *p* = 0.008), as shown in Figure 3A. There were also borderline significant differences suggesting an increased co-activation in the left frontal orbital cortex [(MNI: −36, 27, −22), corrected *p* = 0.08], with participants showing increased activation at post-test compared to baseline within SS06 (please see Figure 3B). Similarly, borderline significantly decreased co-activation in the left frontal lobule/superior frontal gyrus [(MNI: −18, 42, 31), Brodmann Area (BA) 9, corrected *p* = 0.09] within SS23, as shown in Figure 3C. Additionally, a borderline increased co-activation was seen following the exercise program in the left occipital fusiform gyrus/lateral occipital cortex [(MNI: −40, −74, −16), BA 19, corrected *p* = 0.07] within SS30, as shown in Figure 3D. The brain regions identified to be involved in each independent component for the Spatial Span task are reported in Table 3.

**TABLE 3:** Brain regions composing the Spatial Span-independent components (group-level spatial maps) identified via independent component analysis.

**Brain regions**	**MNI coordinates (*x*, *y*, *z*)**	***Z* score**
**SS06**		
Cerebellum	23, −36, −32	11.3
Fontal lobule (BA 10), R	5, 57, 34	9.1
Inferior frontal gyrus, R	56, 28, 25	13.2
Lateral occipital cortex, R	56, −63, −14	10.1
Middle frontal gyrus, R	50, 33, 33	15.1
Precentral gyrus, R	51, 10, 30	13.9
Precentral gyrus, L	−52, −0, 50	9.9
Precuneus cortex, L	−2, −78, 42	10.5
Supramarginal gyrus, R	59, −40, 44	12.5
**SS16**		
Angular gyrus, L	−43, −53, 19	10.7
Inferior frontal gyrus, R	48, 8, 15	10.1
Lateral occipital cortex, L	−23, −89, 13	11.5
Lateral occipital cortex, R	56, −60, 13	13.4
Middle frontal gyrus, R	33, 5, 65	13.1
Occipital pole, R	18, −98, 7	12.6
Precentral gyrus, L	−44, −2, 35	11.2
Superior frontal gyrus, R	22, −6, 74	10.9
Superior parietal lobule, L	−24, −54, 55	10.3
**SS23**		
Cerebellum	42, −51, −49	14.3
Lateral occipital cortex, R	12, −63, 64	9.4
Postcentral gyrus, R	28, −37, 75	9.8
**SS30**		
Central opercular cortex, L	−47, 3, 3	9.8
Inferior frontal gyrus, L	−57, 22, 14	9.4

*SS, Spatial Span-independent components (group-level spatial maps); BA, Brodmann area; L, left hemisphere; R, right hemisphere. Regions are reported as peak of cluster activation (*Z* score) within each component.*

**FIGURE 4:**
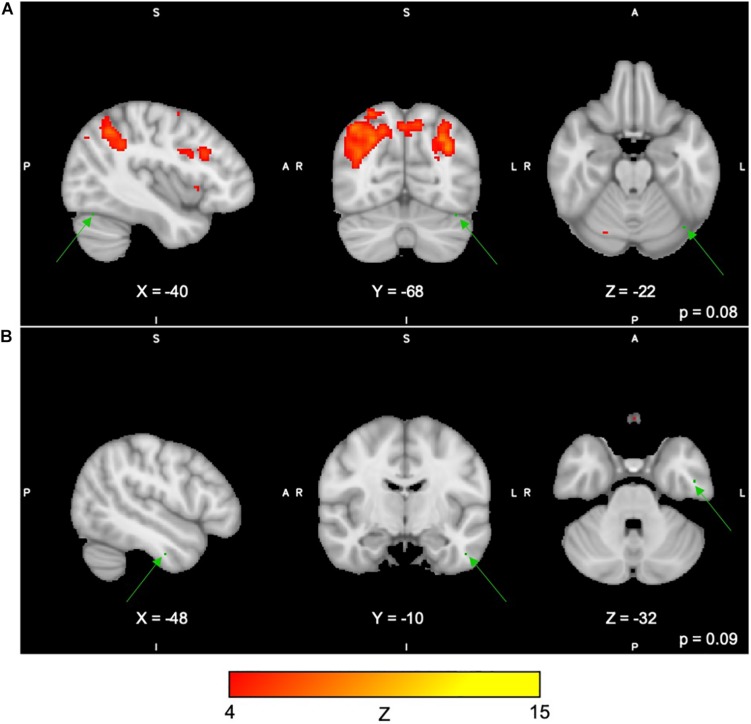
**(A,B)** Changes within group during the Digit Span task. In red–yellow contrast are Digit Span-independent components 06 **(A)** and 08 **(B)**. In dark–light green are regions with changes in group-level spatial map co-activation after the exercise program (green arrows).

For the Digit Span task, there were no significant differences following the exercise program across all 40 group-level spatial maps [i.e. Digit Span-independent components (DS)]. However, borderline significant differences were found in the DS06 in which increased co-activation was seen in the left occipital fusiform gyrus [(MNI: −40, −68, −22), corrected *p* = 0.08] (see Figure 4A). In addition, increased co-activation was seen within the DS08 located in the left inferior temporal gyrus [(MNI: −48, −10, −32), corrected *p* = 0.09] (see Figure 4B). The brain regions identified to be involved in each independent component for the Digit Span task are reported in Table 4.

**TABLE 4:** Brain regions composing the Digit Span-independent components (group-level spatial maps) identified via independent component analysis.

**Brain regions**	**MNI coordinates (*x*, *y*, *z*)**	***Z* score**
**DS06**		
Supramarginal gyrus, R	51, −44, 43	9.7
Supramarginal gyrus, L	−45, −49, 42	9.7
Superior parietal lobule, L	−31, −55, 44	13.2
Lateral occipital cortex, R	31, −64, 58	9.9
Lateral occipital cortex, L	−24, −60, 44	12.3
Angular gyrus, R	45, −57, 44	12.2
**DS08**		
Subcallosal cortex, L	−10, 23, −17	11.9
Frontal medial cortex, R	10, 34, −20	12.5
Frontal medial cortex, L	−7, 38, −17	10.8

*DS, Digit Span-independent components (group-level spatial maps); L, left hemisphere; R, right hemisphere. Regions are reported as peak of cluster activation (*Z* score) within each component.*

**FIGURE 5:**
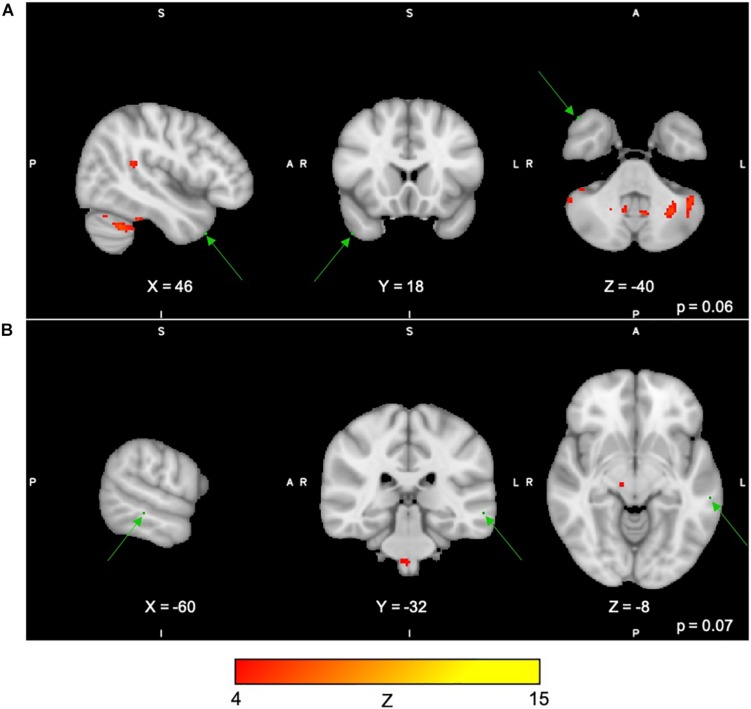
**(A,B)** Changes within group during the Paired Associates task. In red–yellow contrast are IC15 **(A)** and IC08 **(B)**. In dark–light green are regions of decreased co-activation after the exercise program (green arrows).

For Paired Associates task across all 40 group-level spatial maps [i.e. Paired Associates-independent components (PA)], there were no significant differences following the exercise program. However, borderline significant differences were found in PA15 in which increased co-activation was seen in the right temporal lobe [(MNI: 46, 18, −40), BA 38, corrected *p* = 0.07], as well as in PA34, where decreased co-activation was seen in the left middle temporal gyrus [(MNI: −60, −32, −8), corrected *p* = 0.06] following the exercise program (please see Figures 5A,B). The brain regions identified to be involved in each independent component for the Paired Associates task are reported in Table 5.

**TABLE 5:** Brain regions composing the contrast are shown Paired Associates-independent components (group-level spatial maps) identified via independent component analysis.

**Brain regions**	**MNI coordinates (*x*, *y*, *z*)**	***Z* score**
**PA15**		
Cerebellum	−30, −51, 43	17.4
Lingual gyrus, L	−6, −52, −2	10.7
Supramarginal gyrus, R	53, −41, 20	9.7
Middle temporal gyrus, R	52, −48, 6	9.2
**PA34**		
Temporal pole (BA21), L	−25, 3, −36	8.2
Parahippocampal gyrus, L	−24, 1, −36	8.1

*PA, Paired Associates-independent components (group-level spatial maps); L, left hemisphere; R, right hemisphere. Regions are reported as peak of cluster activation (*Z* score) within each component.*

### *Post hoc* Analysis

In our *post hoc* analysis (using dual regression stage 2 outputs), we explored within group-level spatial map by extracting summary values that indicated how strongly the voxels of a given map were associated with the time course for that map (e.g. Spatial Span 16) and whether those values changed over time. This *post hoc* analysis was limited to group-level spatial maps that were significant in our main analysis (i.e. dual regression). We extracted summary values from the entire group-level spatial maps as well as for the specific locations that showed changes over time using MNI coordinates. For example, we looked at the average connectivity change within the right precentral/postcentral gyri (MNI: 36, −22, 58) for SS16 from baseline to 24 weeks.

**FIGURE 6:**
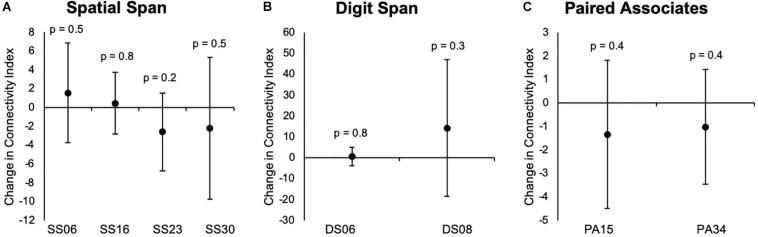
**(A–C)** Changes overtime in the average strength of functional connectivity within each group-level spatial maps for each task that showed significant changes from baseline to 24 weeks in the main analysis. Data are presented as mean difference from baseline to 24 weeks and associated confidence interval, along with *p*-values for significant changes. SS, Spatial Span-independent components; DS, Digit Span-independent components; PA, Paired Associates-independent components.

**FIGURE 7:**
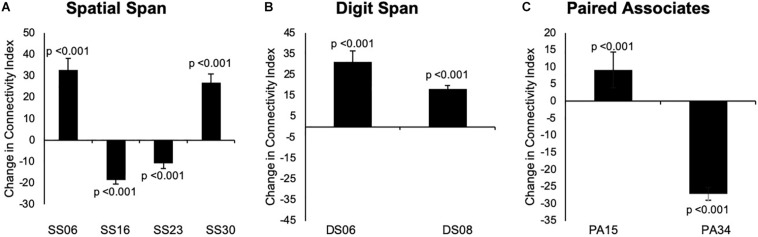
**(A–C)** Changes overtime in the average strength of functional connectivity for the specific region where changes from baseline to 24 weeks in group-level spatial map co-activation were observed. Graph A illustrates changes in the left frontal orbital cortex (MNI: –36, 27, –22) for SS06; right precentral/postcentral gyri (MNI: 36, –22, 58) for SS16, left frontal lobule/superior frontal gyrus (MNI: –18, 42, 31, BA 9) for SS23, and left occipital fusiform gyrus/lateral occipital cortex (–40, –74, –16, BA 19) for SS30. Graph B illustrates changes in the left occipital fusiform gyrus (MNI: –40, –68, –22) for DS06 and left inferior temporal gyrus (MNI: –48, –10, –32) for DS08. Graph C illustrates changes in the right temporal lobe (MNI: 46, 18, –40, BA 38) for PA15 and left middle temporal gyrus (MNI: –60, –32, –8) for PA34. Data are presented as mean difference from baseline to 24 weeks and associated confidence interval, along with *p*-values for significant changes. Note: SS, Spatial Span; DS, Digit Span; PA, Paired Associates-independent components; MNI, Montreal Neurological Institute coordinates; BA, Brodmann area.

Our results indicated that there were no significant changes in group-level spatial maps average FC from baseline to 24 weeks across all three tasks. When only considering the regions where significant or borderline significant changes occurred in the main analysis, we noted changes in the average FC from baseline to 24 weeks, which confirmed the results from dual regression. The results are summarized in Figures 6, 7.

## Discussion

We conducted a secondary analysis of four memory tasks following a 24-week multiple-modality exercise and with or without additional mind–motor training. We also conducted a data-driven exploratory analysis of task-related cortical FC changes as a result of multiple-modality exercise and mind–motor training (M4 group) in older adults with SCC at increased risk for dementia. Following 24 weeks of intervention, we observed significant differences between groups in the Paired Associates tasks, favoring the experimental group, which received additional mind–motor training (i.e. M4 group) compared to the active control group. Further, our exploratory analysis revealed significant and borderline significant changes in FC during three of the four memory tasks administered in our study. Owing to the approach used in our investigation, the results from our fMRI substudy must be interpreted within the context of each task and each independent component derived from the ICA. Our analysis was aimed at exploring within-group spatial map FC changes after the intervention. Using MELODIC ICA, we were able to identify independent components that included brain regions that were temporally associated (i.e. co-activation) during each task and, therefore, could be understood as functionally associated (Dosenbach et al., 2007; Biswal et al., 2010). It is relevant to note that some of the regions that also co-active during a task (temporally, but not functionally correlated) might not necessarily be a result of task-related processes, but rather the result of other neuronal processes concurrent to task performance (Hampson et al., 2006). With these considerations, it is then possible to question whether the intervention had any impact within the FC of the brain for a given task in our study.

Overall, the results from our full sample suggested that additional mind–motor training yielded greater changes in memory measured in the Paired Associates task superior to multiple-modality exercise without mind–motor training, with trends for significant changes in the Digit Span task at the follow-up. Results from our exploratory, data-driven fMRI analysis indicated that our experimental condition might have imparted divergent effects on cortical FC across the tasks employed; however, the results must be considered with caution. More specifically, for the Spatial Span task, we observed decreased co-activation in the precentral/postcentral gyri (corrected *p* = 0.008) and left frontal lobe/superior frontal gyrus (trend), as well as increased co-activation in the left frontal orbital cortex and left occipital fusiform gyrus/lateral occipital cortex (trend). For the Digit Span task, we observed increased co-activation in the left occipital fusiform gyrus and left inferior temporal gyrus (trend). Last, for the Paired Associates task, we observed increased co-activation in the right temporal lobe (trend) and decreased co-activation in the left middle temporal gyrus (trend). Our *post hoc* analysis investigating changes in FC strength across the entire group-level spatial maps following previous methodology (Glahn et al., 2010) revealed no significant differences following the program. Although, when exploring each specific cortical region within the group-level spatial maps for connectivity strength, we encountered statistical significance, suggesting a confirmation of the changes in the co-activation in the spatial maps (please see Figure 7).

Across all four tasks, significant changes were seen only for the Spatial Span task in the right precentral/postcentral gyri. For this task, we observed a decreased co-activation within the group-level spatial maps from baseline to 24 weeks. The group-level spatial map (SS16) in which this change occurred involves co-activation of brain regions previously associated with executive control (e.g. superior parietal lobule), working memory (e.g. superior frontal gyrus), as well as sensorimotor and visuospatial areas (Shirer et al., 2012). In the context of this group-level spatial map, it is possible to suggest that the decreased FC of the precentral/postcentral gyri with the other cortical regions did not have an imperative effect on task performance at 24 weeks, owing to the fact that there were no significant changes in the behavioral scores for the Spatial Span task for our full sample, nor for our subsample in this M4 group.

The Spatial Span task is believed to measure spatial short-term memory ability (Kessels et al., 2000). It is noteworthy that our program included a 15-min block of SSE, in which participants are expected to memorize and reproduce increasingly complex stepping patterns on a gridded floor map (Shigematsu et al., 2008). Arguably, the SSE program demands increased attention and short-term spatial memory recall, which could lead to improvements in overall spatial memory performance. Although speculative, it is possible that the SSE program, in addition to the multiple-modality exercise program, could have yielded FC changes involving co-activation of the precentral/postcentral gyri during Spatial Span task performance in the current study. This could be further supported by trends of increased co-activation observed in the left inferior temporal gyrus during another task in this study, the Digit Span task, a region engaged in motor function and known to show decreased connectivity in older adults compared to young individuals in resting-state fMRI (Voss et al., 2013). Because of methodological limitations, these interpretations must be interpreted with caution.

It is, however, undoubtedly challenging to attribute changes in FC of motor-related regions (i.e. precentral/postcentral gyri and left inferior temporal gyrus) during computer-based memory tasks to the effects of our program, since we are unable to establish a direct connection between changes in the co-activity and task performance, in addition to estimating region engagement from resting to task-related states (Hampson et al., 2006). Because of a lack of significant changes in the behavioral measures for the Spatial Span and Digit Span tasks (trend for significant changes in the full sample), it is also difficult to suggest whether increases in co-activation would indicate negative changes in FC due to aging or disease-related processes and/or whether decreases in co-activation would indicate efficiency during task performance due to the intervention applied in our study. Moreover, as mentioned above, these processes could also be considered task irrelevant, which might or might not be detrimental to task performance (Hampson et al., 2006). In addition, a previous study did not observe changes in FC of the motor regions following 6 and 12 months of AE in older adults (Voss et al., 2010). Voss et al. (2010) reported that the exercise program did not lead to any changes in regional FC in motor areas such as the right precentral gyrus and left inferior temporal gyrus. There is evidence from animal literature suggesting brain plasticity identified as increased synaptic density and expression of proteins associated with dendritic growth in motor-related regions following treadmill exercise (Swain et al., 2003; Ferreira et al., 2010), and even more so with more complex motor training (Garcia et al., 2012).

Therefore, in our limited design, we cannot determine with certainty if the task-related FC changes observed in our study are due to the intervention itself and whether these are positive meaningful changes. In the context of previous studies adopting a similar data analysis methodology, Chirles et al. (2017) investigated FC changes in older adults diagnosed with MCI following a 12-week AE program (Chirles et al., 2017). The authors were mainly interested in exploring FC of the posterior cingulate cortex and precuneus within the DMN. The authors reported an increased co-activation in resting-state FC between the posterior cingulate cortex and precuneus regions and the several other cortical regions, including the right postcentral gyrus. This suggested that the aerobic program enhanced recruitment of preserved brain regions in MCI patients, which possibly reflected in improvements in behavioral measures of cognitive function. The FC improvements were not seen in the healthy control group—despite improvements in behavioral measures in these participants (Chirles et al., 2017).

Noteworthy, we reported borderline significant changes (confirmed in our *post hoc* region-specific analysis) in FC in the right temporal lobe (BA 38) and left middle temporal gyrus during the Paired Associates task, two regions heavily involved in memory processes (Shirer et al., 2012). Moreover, our behavioral data showed greater changes in the Paired Associates tasks for our full sample analysis and also borderline significant changes in the task performance in our subsample. Under these considerations, we can postulate that our multiple-modality exercise and mind–motor training program might have had a positive effect in FC underlying visuospatial memory, as measured by improved performance in the full sample, and in our nine participants from the M4 group (trend at *p* = 0.08) in the Paired Associates task with a medium-to-large effect size (i.e. Cohen’s d for max score = 0.72 and 0.74 for mean score) (Supplementary Table 2). More importantly, the results from our full sample analysis revealed that there were indeed significant improvements in the Paired Associates task performance above and beyond the active control group (*p* = 0.001 for changes overtime and *p* = 0.019 for difference between groups at 24 weeks). The data from our full sample offers confirmation and strengthens our borderline significant changes in the Paired Associates behavioral data within our subsample, which can then provide context and assist in interpretation of the borderline significant changes in FC observed in this fMRI sub-study.

Cortical regions involved in the group-level spatial maps where the FC changes occurred, that is, independent components PA15 and PA34 (please see Table 5), were predominately located in the medial temporal lobe, including the left and right hippocampi, parahippocampal gyri, and middle temporal gyrus (please see Supplementary Figures 2A,B). It is well-known that these regions have been implicated in memory function (Alvarez and Squire, 1994; Jeneson and Squire, 2011; Shirer et al., 2012), and have been observed to be heavily involved in the Paired Associates task memory encoding and retrieval (De Rover et al., 2011). From a clinical perspective, these findings could have important implications, considering that these aforementioned regions are hallmarks of pathophysiological changes (e.g. amyloid beta deposition) in MCI and early/prodromal stages of Alzheimer’s disease (Taylor and Probst, 2008), including cortical atrophy proceeding Alzheimer’s disease diagnosis (Pettigrew et al., 2017), and disruption of resting-state FC, possibly due to Alzheimer’s disease biomarker deposition (Song et al., 2015). Moreover, the performance on a variant of the Paired Associates task employed in this study demonstrated marked differences between MCI patients and healthy controls, characterized by decreased bilateral hippocampal and parahippocampal activation during tasks in MCI patients compared to controls (De Rover et al., 2011).

Here, we were able to demonstrate significant changes in the behavioral component of memory function measured via the Paired Associates task. This is an encouraging result, and future research could investigate the effects of multiple-modality exercise and mind–motor training in medial temporal lobe regions, employing a full RCT design and including resting-state and task-related FC as the main outcomes. It would be relevant to use a task such as the Paired Associates task to explore such effects, as postulated by De Rover et al. (2011), regarding the relevance of the task as a possible biomarker of Alzheimer’s disease risk (De Rover et al., 2011).

### Limitations

Our findings should be interpreted with caution and in the context of our limitations. Although the CBS is grounded in well-validated neuropsychological tests (Hampshire et al., 2012), this is the first study to apply this method to evaluate the effects of exercise in memory function in older adults with SCC. Also, participants included in this study were predominantly Caucasian, well educated, and functionally independent; thus, our results may not be generalizable to other populations. For our exploratory fMRI substudy, our data analysis was restricted to nine subjects only, a very small sample size, limiting our ability to generalize the results. We had limited resources to collect fMRI data from our active control group, and therefore, we cannot establish certainty on whether our findings were due to the main effects of the intervention program—even though the results from the experimental group in our full data analysis of memory tasks showed greater changes in memory following the program (driven by changes in the Paired Associates task), superior to the active control group. In addition, we did not include resting-state data in our study, impairing our ability to determine which regions identified in the task-derived independent components were, in fact, relevant to the task performance or were a result of other processes irrelevant to the task performance (Hampson et al., 2006). Importantly, our group-level results were also susceptible to artifacts, and the group-level maps could have included regions in which co-activation was seen due to noise, despite our efforts to correctly identify and remove artifact-driven independent components at the individual level. In addition, despite our efforts to mitigate sources of noise and variability influencing the BOLD response, we acknowledge that this is still a possibility. However, it is unlikely that the individual-level and group-level maps would significantly suffer from, or be heavily influenced by, the variability of BOLD response or non-task processes, as BOLD variability would be a product of random noise, and not a specific pattern equally present in all individuals during assessment.

Another limitation of our study was that we adopted a model-free approach to analyze the task-related fMRI data (Rogers et al., 2007). We used this approach to investigate whole-brain, voxel-wise FC maps that could have been active for the duration of each task, and therefore, our methods were restricted to data-driven exploratory analysis as opposed to a hypothesis-driven approach (where previous knowledge would have informed the decision of limiting analysis to a set of cortical and subcortical regions of interest) (Rogers et al., 2007). In addition, our model-free design only allowed us to collect data during a single 5-min block of ongoing trials for each task, and consequently, we were not able to time lock the stimulus and data collection of each single trial within the block (as commonly done in event-related or block design studies). This could have ultimately reduced our power to detect true significant treatment effects (Huettel, 2012).

In addition, FC data is particularly sensitive to head motion and physiological artifacts linked to respiratory and cardiac rhythms (Buckner et al., 2013). Furthermore, the FC data provide essential insights into the cortical and subcortical coupling at rest and during task-related fMRI; however, it is unknown whether the observed FC within a group-level spatial map in this study reflects stable or temporary connectivity configurations in the brain (Buckner et al., 2013). Finally, our study employed a multi-domain intervention, involving components of aerobic training, resistance training, as well as mind–motor training. This is a novel approach, and to our knowledge, no previous neuroimaging studies have been conducted to investigate changes in FC during memory tasks in older adults with SCC following a multi-domain program such as ours. Therefore, methodological differences between our study and previous studies create a barrier to draw conclusions regarding our results.

## Conclusion

Our aim was to explore the effects of 24 weeks of multiple-modality exercise with or without additional mind–motor training in four memory tasks, and explore task-related, cortical and subcortical FC changes in older adults with SCC. Our findings indicated that multiple-modality exercise with additional mind–motor training yielded greater changes in memory function during the Paired Associates task compared to an active control group. Further, our intervention might have resulted in divergent FC adaptations, including significant decreased co-activation in the precentral/postcentral gyri during the Spatial Span task. Of particular interest, we also reported borderline significant increased co-activation in the right temporal lobe, accompanied by a decreased co-activation in the left middle temporal gyrus within the two group-level spatial maps involving regions of the medial temporal lobe during the Paired Associates task. These findings provide insight into the potential of our multiple-modality exercise and mind–motor training intervention to promote improvements in behavioral measures of visuospatial memory, as well as impart FC adaptations in brain regions relevant to Alzheimer’s disease risk. Future research should emphasize the clinical relevance of these FC changes following exercise in the context of disease prevention and treatment.

## Author’s Note

This work was conducted at the Parkwood Research Institute, in affiliation with the Lawson Health Research Institute and St. Joseph’s Health Care (London, ON, Canada).

## Data Availability Statement

The datasets generated for this study are available on request to the corresponding authors.

## Ethics Statement

The studies involving human participants were reviewed and approved by the Western University Health Sciences Research Ethics Board. The patients/participants provided their written informed consent to participate in this study.

## Author Contributions

NB was responsible for the study design, data preprocessing and analysis, interpretation of results, and drafting of the final manuscript. LN was responsible for the data preprocessing and analysis, interpretation of results, and reviewing and editing the manuscript. DG was responsible for the study design, data collection, and reviewing of the manuscript. AO was responsible for the study design and reviewing and editing the manuscript. RP acquired funding for the study, was responsible for the study design, data collection, interpretation of results, and reviewing and editing the manuscript.

## Conflict of Interest

The authors declare that the research was conducted in the absence of any commercial or financial relationships that could be construed as a potential conflict of interest.

## Footnotes

1 www.fmrib.ox.ac.uk/fsl
