# Supplementary material for: Memory Function and Brain Functional Connectivity Adaptations Following Multiple-Modality Exercise and Mind–Motor Training in Older Adults at Risk of Dementia: An Exploratory Sub-Study
Source: Front Aging Neurosci. 2020 Feb 25;12:22. doi: 10.3389/fnagi.2020.00022 (PMC7052336; doi:10.3389/fnagi.2020.00022)
Supplement: Supplementary file 1 [file Data_Sheet_1.pdf]

Supplementary Material

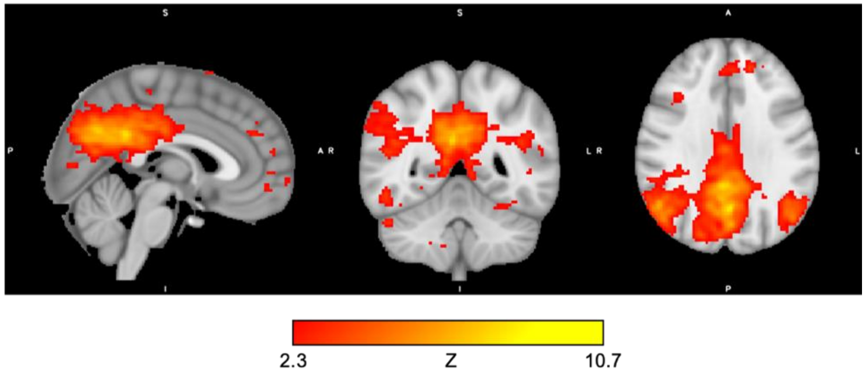

Supplementary Figure 1. Default mode network (DMN) identified via group independent component analysis (ICA) during Monkey Ladder task.

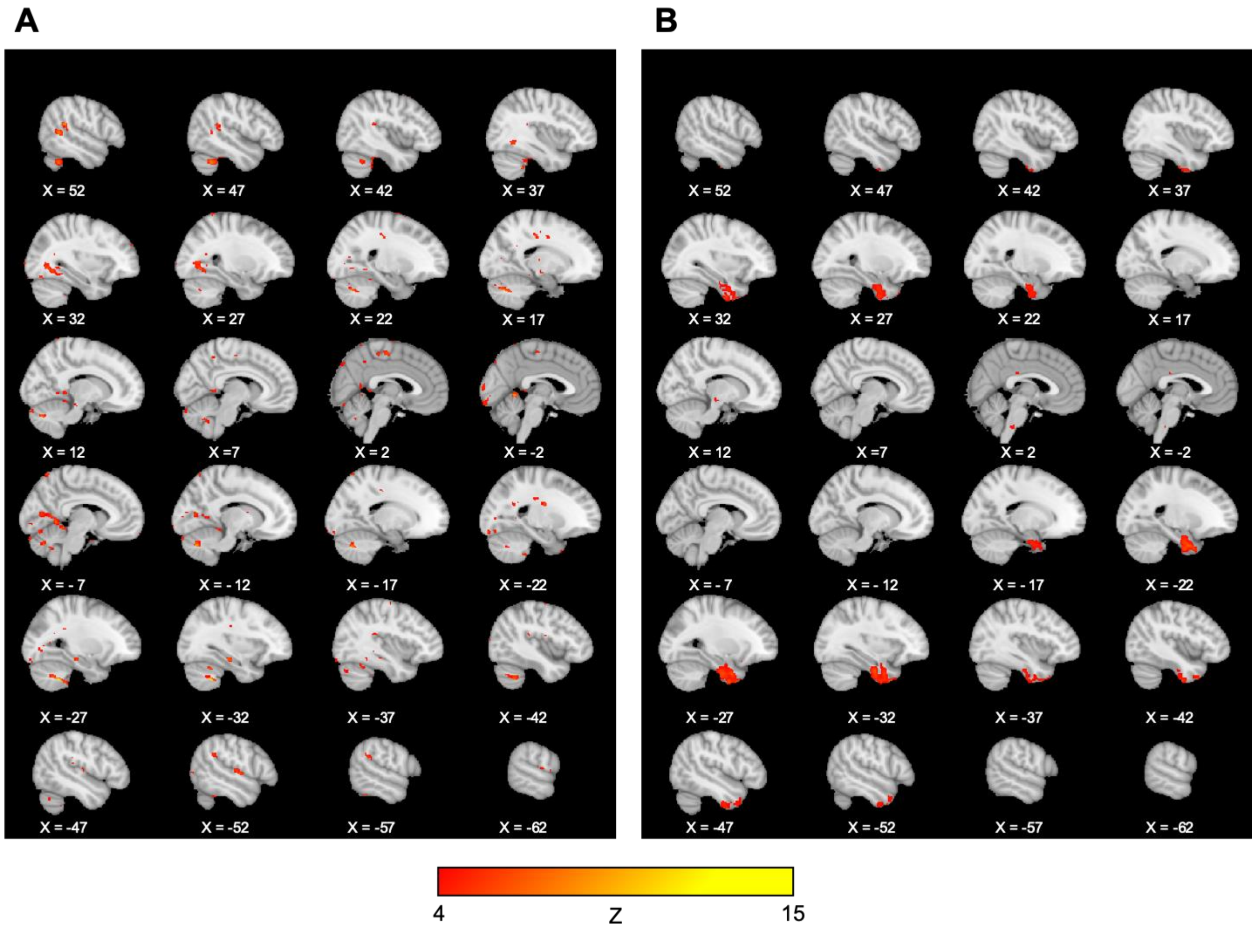

Supplementary Figure 2. Group-level spatial maps (A = PA15; B = PA34) identified via independent component analysis (ICA) during Paired Associates task.

Supplementary Table 1. Baseline demographic and clinical characteristics.

Variables	Study sample (n = 9)
Demographics	
Age, yr	67.8 (8.8)
Women, *n*	8
Caucasian, *n*	9
Education, yr	14.1 (3.1)
MoCA, score	24.9 (2.9)
MMSE, score	29.2 (0.7)
Hypertension, *n*	5
Hypercholesterolemia, *n*	2
Type 2 diabetes, *n*	1

Data presented either as mean (standard deviation) or *n* where applicable.

Note: MMSE, Mini-Mental Status Examination; MoCA, Montreal Cognitive Assessment.

Supplementary Table 2. Changes in behavioral cognitive tasks from baseline to 24 weeks.

Behavioral tasks	Baseline	24 weeks	Differences from baseline (95% confidence interval)	*t* Value	*p* Value	Cohen's *d*
Monkey Ladder †						
Max score	5.8 (.7)	6 (1.1)	0.3 (-0.3 to 0.8)	1	0.35	0.35
Mean score	4 (0.5)	4.4 (0.6)	0.4 (-0.1 to 0.9)	1.7	0.12	0.62
Spatial Span †						
Max score	4.4 (1.3)	4.3 (0.9)	-0.1 (-1.3 to 1)	-0.3	0.80	-0.09
Mean score	3.5 (0.9)	3.5 (0.8)	0.01 (-0.8 to 0.8)	-0.02	0.99	-0.01
Digit Span						
Max score	6.2 (1.6)	6.3 (1.1)	0.1 (-0.4 to 0.6)	0.6	0.59	0.18
Mean score	5.1 (1.2)	5 (0.9)	-0.1 (-0.5 to 0.3)	-0.7	0.49	-0.24
Paired Associates †						
Max score	3.9 (0.8)	4.6 (0.9)	0.8 (-0.1 to 1.6)	2	0.08	0.72
Mean score	2.9 (0.4)	3.3 (0.6)	0.4 (-0.1 to 0.8)	2.1	0.08	0.74

Behavioral tasks are expressed in arbitrary units. Data presented as mean (standard deviation) or otherwise indicated. †Behavioral data missing for 1 participant in each task.
